# A descriptive study of 16 severe *Plasmodium vivax* cases from three municipalities of Colombia between 2009 and 2013

**DOI:** 10.1186/1475-2875-13-404

**Published:** 2014-10-15

**Authors:** Anthony T O’Brien, Jesica F Ramírez, Sandra P Martínez

**Affiliations:** Fundación Santa Fe de Bogotá Centro de Estudios e Investigación en Salud - CEI, Carrera 7 B # 123-90, Piso 3, Bogotá, Colombia

**Keywords:** *Plasmodium vivax*, Colombia, Severe

## Abstract

**Background:**

*Plasmodium vivax,* the most geographically distributed cause of malaria, accounts for more than 70% of cases in the Americas. In Colombia, *P. vivax* was responsible for 67.3% of cases in the last five years. Despite vivax malaria impact worldwide, historically it has been neglected and considered to be a benign disease. In the last decade medical literature reports have emerged countering this benign outlook*.* This study pretends to describe the clinical and paraclinical profile of severe vivax malaria cases hospitalized in Tumaco, Cali, Buenaventura between 2009 and 2013, to contribute to the knowledge regarding the behaviour and clinical expression of this disease.

**Methods:**

This is a descriptive, retrospective case-series study of 16 severe malaria vivax cases, hospitalized between 2009 and 2013, in Colombian municipalities of Tumaco, Buenaventura and Cali. Severe malaria vivax cases were defined using criteria adapted from the national guidelines. Descriptive analyses of reason for consultation, signs and symptoms, diagnosis, treatment, paraclinical characteristics, complications, and time hospitalized, were conducted.

**Results:**

Sixteen cases of severe *P. vivax* were analysed. Fever, chills and headache were shown to be the main admission symptoms. Elevation of total bilirubin levels in 18.75%, and severe thrombocytopaenia in 25% of cases were the main complications presented during hospitalization. All cases responded to treatment, there were no deaths.

**Conclusions:**

The following questions derived from this study could be the basis for future research: 1) Does the time to consultation have an impact on the number of days hospitalized and how cases progress during hospitalization, 2) Are the severity criteria in WHO guidelines sensitive enough to be used in clinical practice compared to national guidelines, and 3) How does malnutrition contribute to anaemia in malaria-endemic regions.

## Background

Malaria, one of the most important parasitic diseases of the 21^st^ Century, is caused by parasites of the genus *Plasmodium* [1]. The disease significantly impacts global health; in 104 malaria-endemic countries where an estimated 207 million cases (135–287 million) and an estimated 627,000 deaths (473,000-789,000) have been reported in 2012 [2].

Currently *Plasmodium vivax* is the most geographically-distributed cause of malaria worldwide. Although, estimates vary, it is projected that 2.85 billion people live at risk [3], and annual infections range between 132–391 million [4, 5]; in the Americas, about 5.5% of people are at-risk [6], and *P. vivax* accounts for more than 70% of cases [7]. Furthermore, in the Americas about 30% of the population of the 21 countries with active transmission are at some degree of risk; Brazil and Colombia accounted for 68% of the cases in 2011 [2].

In Colombia, approximately 27 million individuals live in areas suitable for transmission [8], and, although the mortality of the disease has diminished, the associated morbidity has remained relatively constant, with a national registry of 378,764 cases, of which 1,800 were severe in the last five years, whereas *P. vivax* was responsible for 67.3% of all cases [2, 9, 10].

Tumaco (Nariño state), Buenaventura and Cali (Valle state) on the Pacific coast, are the most representative sites for high intensity transmission [8]. Buenaventura is at 7 metres above the sea level, average temperature 27°C, 6,500 mm annual rainfall. Cali is located at an altitude of 995 metres above sea level, on average the temperature is 23°C, the average annual precipitation is 1,000 mm in most of the metropolitan area, Tumaco is located 3 metres above sea level the average temperature ranges between 26 to 28°C, [11–13]. Buenaventura, Cali and Tumaco, have unique characteristics that make the transmission and proliferation of Plasmodium species more prominent, and are considered endemic areas of malaria.

Despite vivax malaria significant contribution towards the overall case count worldwide, historically it has been neglected and considered to run a benign disease course compared to falciparum malaria. This has led to reduced interest in research, funding, control strategies, health and socio-economic impact evaluation [5, 14–17].

In the last decade, increasing reports in medical literature have emerged to globally counter the benign outlook on *P. vivax*, documenting clinical outcomes perceived to be principally of *P. falciparum*, including cerebral malaria, hydrocephalus, retinal haemorrhage, myocarditis, acute respiratory distress, pulmonary oedema, gastro-intestinal symptoms, jaundice, hepatic dysfunction, acute kidney injury, severe anaemia, neutropaenia, thrombocytopaenia, haemoglobinuria, hypoglycaemia and shock [17–24].

Data supplementing and interpreting the increase in severe vivax malaria cases are scarce, additionally, caution is required due to weighty contributory factors, such as its relapsing nature due to hypnozoites, incidental parasitaemia, co-morbidities, treatment resistance, and access to opportune healthcare [22, 25].

As of 19^th^ March 2014, the search within the US National Library of Medicine National Institute of Health database for severe malaria due to *P. falciparum* retrieved almost seven times more abstracts than for severe malaria due to *P. vivax*. This situation highlights the urgency to investigate in depth severe malaria cases due to *P. vivax*.

Therefore, this study intends to establish the clinical and paraclinical profile of severe malaria vivax cases hospitalized in Tumaco, Cali, and Buenaventura between 2009–2013, to contribute to the knowledge regarding the behaviour and clinical expression of this disease.

## Methods

### Study design

This is a descriptive, retrospective case-series study of 16 severe malaria vivax cases, hospitalized between 2009 and 2013. The data were mined from medical records revised at four hospitals located in endemic areas of the Colombian states of Nariño and Valle del Cauca, covering the municipalities of Tumaco, Buenaventura and Cali.

Medical doctors were employed and trained in order to fill out a virtual instrument accessible via email. The instrument included the following information: personal identification, past medical history, history of present illness, clinical characteristics, physical examination, diagnosis, paraclinical and parasitological results on admission, treatment and case outcome.

To assure that the instrument was filled out correctly, three independent reviewers evaluated the quality of the information filled out for each medical report and provided feedback when necessary to correct discrepancies. This was carried out at least twice, before and after feedback.

Frequencies, central tendency and dispersion were calculated. Median and comparison of frequencies was used to analyse the data. Descriptive analysis was processed using STATA® version 12.

Variables considered in the descriptive analysis were: gender, age, place of origin, duration of symptoms before admission, symptomology on admission, presence of co-morbidities, days hospitalized, vital signs on admission, thick and thin blood smear slide results, physical examination on admission, medical treatment received, initial laboratory values, parasitaemia, complications during hospitalization, pregnancy status, previous history of malaria, and prior medical attendance before admission.

### Case definition of severe vivax malaria

Subjects hospitalized between 2009–2013, with diagnostic confirmation of *P. vivax* via thick blood smear slides, and with one or more of the clinical or paraclinical criteria mentioned in the Colombian national guidelines for severe malaria, were defined as cases (Table 1).

**Table 1:** **Criteria used to define severe malaria cases according to national guidelines**

Clinical criteria*	Laboratory criteria*
1. Loss of consciousness or profound coma	1. Haemoglobinuria
2. Prostration; extreme weakness with inability to walk or sit without assistance	2. Hypoglycaemia (<60 mg/dl)
3. Inability to feed oneself	3. Metabolic acidosis (plasmatic bicarbonate <15 mmol/L)
4. Multiple seizures; more than 1 episode in 24 hours	4. Hyperlactaemia (lactate acid <5 mmol/L)
5. Respiratory distress syndrome	5. Severe anaemia (haemoglobin <7 g/dL, haematocrit <21%)
6. Circulatory collapse/shock; systolic arterial pressure <80 mmHg in adults and <50 mmHg in children	6. Hyperparasitaemia (>50,000 asexual parasites/uL) with the diagnosis of *P. falciparum*, or mixed infection with *P. vivax*
7. Clinical jaundice with signs of vital organ failure	7. Thrombocytopaenia (<50,000 mm^3^)
8. Spontaneous haemorrhage	8. Elevated transaminase (>80 IU)
9. Pulmonary oedema evidenced through radiography	9. Elevated total bilirubin (>1.5 mg/dL)
	10. Renal insufficiency (serum creatinine level >1.5 mg/dL)

*Ministry of Social Protection. Republic of Colombia. Public Health General Management. National Institute of Health. World Health Organization: Guideline for the integral clinical treatment of patients with Malaria. Bogotá; 2010 [41].

The Colombian National guidelines for severe malaria define hepatic dysfunction as transaminase values >40 IU. According to medical literature and the WHO malarial hepatic dysfunction is defined as an elevation of three times the upper limit of normal transaminase values [26, 27]. An intermediate value of >80 IU was utilized in this study [27–29].

### Exclusion criteria

Cases with sickle cell disease, viral or bacterial infections and/or other diseases that could explain the clinical presentation were excluded, as well as cases that originated from a municipality not included within the area of analysis, or cases that were transferred from another municipality to receive medical care.

Ethical approval for access to patient medical history data and extraction for analysis and conversion to scientific manuscript was approved by the Ethical Committee of Hospital Fundación Santa Fe de Bogotá.

## Results

### Demographic characteristics

In total there were 16 cases of severe vivax malaria identified, 11 cases were from the municipality of Tumaco, three cases from Cali and two cases from Buenaventura. The median age was 23.5 years (range: five to 57 years). Demographic characteristics regarding the 16 cases are further detailed in Table 2.

**Table 2:** **Sociodemographic characteristics of the 16 severe vivax malaria cases**

Age (years)	n (%)
2 to 9	2 (12.50)
15 to 19	4 (25.00)
20 to 29	6 (37.50)
30 to 39	1 (6.25)
40 to 49	2 (12.50)
≥ 50	1 (6.25)
**Sex**	
Male	9 (56.25)
Female	7 (43.75)
**Geographic region**	
Tumaco	11 (68.75)
Buenaventura	2 (12.5)
Cali	3 (18.75)
**Year**	
2009	2 (12.50)
2010	2 (12.50)
2011	2 (12.50)
2012	4 (25.00)
2013	6 (37.50)
**Healthcare affiliation**	
Subsidized	13 (81.25)
Vinculated	1 (6.25)
Contributive	2 (12.5)
**Occupation**	
Homemaker	4 (25.00)
Student	2 (12.50)
Driver	1 (6.25)
Street vendor	1 (6.25)
Manucurist	1 (6.25)
Not available	7 (43.75)

### Past medical history

With regard to existing co-morbidities, one case had a medical history of secondary arterial hypertension, non-specified diabetes mellitus, and cerebrovascular disease. Fifty percent (8/16) of the cases had no previous history of malaria or blood transfusion in the previous month, and there was no data available for the remaining eight cases. Before reaching a site where medical care was available, 18.8% (3/16) of the cases did self-medicate. Of the three cases that self-medicated, two used paracetamol and one was unclear about which medication was used. Two of the cases were pregnant women.

### Signs and symptoms on admission

The median period of time with symptoms before medical attendance was five days (range: three to 31 days). Fever and chills were the most ubiquitous symptoms, present in all cases. Headache, myalgia, arthralgia, asthenia and adynamia were the following most frequent symptoms as mentioned in Table 3. Dry cough was only present in 6.3% (1/16), while vomiting, was present in 25% (4/16) and abdominal pain in 25% (4/16). Urological symptoms were present in two cases. One case presented polyuria and dysuria, and one case presented dark urine. Haemorrhagic symptoms were alluded to in 12.5% (2/16) cases, one of the cases presented haematochezia and in the other case epistaxis. There were no referred severe neurological, otorhinolaryngological, or constitutional symptoms.

**Table 3:** **Description of cases**

			Vital signs on admission				Physical examination on admission				
Cases	Reason for consultation	Diagnosis on admission	Temperature (°C)	Pulse (BPM)	Respiratory rate (RPM)	Arterial Pressure (Systolic/Diastolic mmHG)	Hydrated	Jaundice	Hepatomegaly	Splenomegaly	Complications during admission and hospitalization
1	Fever, chills, myalgia, asthenia, adynamia	Vivax malaria	36.0	70	20	110/70	Yes	No	No	No	Elevated transaminase
2	Fever, chills, headache, myalgia	Vivax malaria	37.6	76	22	100/90	Data not available	Si	Si	No	Acute renal failure, elevated bilirubin, jaundice with evidence of organ failure
3	Fever, chills, headache, myalgia	Dengue with alarm signs	37.0	100	20	100/80	No	No	No	No	Spontaneous bleeding, elevated transaminase and bilirubin
4	Fever, chills, headache, myalgia	Vivax malaria	39.2	92	29	100/50	Data not available	No	Si	No	Elevated bilirubin
5	Fever, chills, headache	Vivax malaria	36.3	98	20	90/60	No	No	Data not available	Data not available	Shock, thrombocytopaenia
6	Fever, chills, headache, myalgia, asthenia, adynamia	Febril syndrome	39.0	140	24	Data not available	No	No	Data not available	Data not available	Hypoglycaemia
7	Fever, chills, headache, myalgia	Vivax malaria	37.4	84	20	110/70	No	Si	No	No	Elevated transaminase, jaundice with evidence of organ failure
8	Fever, chills, headache,	Febril syndrome	39.0	104	32	60/20	No	No	No	No	Shock, thrombocytopaenia
9	Fever, chills, myalgia, asthenia, adynamia	Vivax malaria	34.5	88	22	110/80	Yes	No	No	No	Thrombocytopaenia
10*	Fever, chills, headache, asthenia, adynamia	Febril syndrome	37.0	100	22	100/60	Yes	No	No	No	Elevated bilirubin
11	Fever, chills, asthenia, adynamia	Vivax malaria	Data not available	80	19	110/70	Yes	Si	No	No	Elevated bilirubin, jaundice with evidence of organ failure
12	Fever, chills, headache, asthenia, adynamia	Vivax malaria	36.5	90	18	100/50	Yes	Si	Si	Si	Shock
13	Fever, chills, headache, asthenia, adynamia	Vivax malaria	37.0	88	22	120/80	Yes	Si	Si	No	Thrombocytopaenia
14	Fever, chills, headache	Vivax malaria	37.0	117	28	Data not available	Yes	No	Si	Si	Spontaneous bleeding, severe anaemia
15	Fever, chills, headache, myalgia	Febril syndrome	39.5	80	19	110/70	No	No	No	No	Elevated bilirubin
16*	Fever, chills	Gestational hypertension	Data not available	84	16	140/90	Yes	No	No	No	Severe anaemia

*Pregnant cases.

At the time of admission all the cases were alert (16/16). Out of the 16 cases, six were dehydrated, eight were hydrated and there is no register for two cases. Adequate diuresis was annotated in eight cases; data were not available for the remaining eight cases. Oral tolerance was registered in 93.75% of cases, data were not available for the remaining cases; it was also confirmed that in the 24 hours prior to the physical examination no case presented more than five emetic or diarrhoeic episodes. Out of the 16 cases, four presented hepatomegaly, and two cases had splenomegaly. Jaundice was found in five cases. No signs of severe neurological or pulmonary compromise were evident.

### Vital signs on admission

The median body temperature was 37.0°C (range: 34.5-39.5°C), the systolic/diastolic pressures ranged between 60/20 and 140/90 mmHg, with a median of 105/70. Out of the 16 cases, the median heart rate was 89.0 BPM (range: 70–140 BPM), and the median respiratory rate was 21.0 RPM (range: 16–32 RPM). Further information about the description of vital signs on admission is presented in Table 3.

### Malaria diagnosis

Methods used to diagnose malaria involved peripheral film, both thick and thin blood smear. No other form of diagnosis techniques for malaria were utilized. *P. vivax* was identified for all cases (16/16). One case was diagnosed with thin blood smear while 93.8% (15/16) were diagnosed with thick blood smears. Parasite count was registered for 15 out of 16 cases. Non-specified parasite count median was 6,720 p/uL (range: 320–36,500 p/uL).

On entry a diagnostic impression of vivax malaria was made for 62.5% (10/16), 25% (4/16) of the cases were thought to be febrile syndrome of unknown origin, 6.3% (1/16) gestational hypertension, and 6.3% (1/16) was suspected to be dengue with warning signs.

### Paraclinical results on admission

All cases were evaluated with a haemogram at the time of admission. Haemoglobin median was 10.7 g/dL (range: 6.2-15.0 g/dL;), haematocrit median was 34.2% (range: 18.1-43.6%), platelets median value was 91,500 mm^3^ (range: 25,000.-328,000 mm^3^). leucocytes median value was 5,935 mm^3^ (range: 2,500-10,800 mm^3^), while median for lymphocyte and neutrophil count was 37.55% (range: 6.0-69.4%) and 49.6% (range: 12.5-94.0%), respectively.

Due to the retrospective nature of the study, register of renal function tests, liver function tests, and other paraclinical test are not available for all the cases. Glycaemia was measured on entry in 56.3% (9/16) cases, transaminases were measured in 50% (8/16), total bilirubin was measured in 25% (4/16) cases, creatinine was measured in 81.25% (13/16) cases and uri-analysis was undertaken in 68.75% of cases (11/16).

The median blood glucose level was 109 mg/dL (range: 74–169 mg/dL), the median value for total bilirubin was four mg/dL (range: 2–5.6 mg/dL), alanine aminotransferase (ALT) and aspartate aminotransferase (AST) median value was 24 IU (range: 10–381 IU) and 29 IU (range: 21–377 IU) respectively, creatinine median value was 0.9 g/dL (range: 0.5-1.6 g/dL). Out of the 11 urinalysis registered, nine were normal, one case had proteinuria and another case presented haematuria. Haemoglobinuria was not observed in any of the cases.

Further information about paraclinical results can be found in Table 4.

**Table 4:** **On admission paraclinical results**

Cases	Haemoglobin (g/dL)	Haematocrit (%)	Platelets (mm3)	Leucocytes (mm3)	Lymphocytes (%)	Neutrophils (%)	Alanine transaminase (UI)	Aspartate aminotransferase (UI)	Creatinine (g/dL)
1	13.65	41.71	120,000	5,370	54.50	27.90	Data not available	Data not available	1.00
2	14.60	43.60	169,000	8,880	20.00	74.70	38.00	32.00	1.60
3	12.10	34.30	82,000	8,100	38.50	41.00	381	377	0.50
4	8.80	Data not available	73,500	10,800	29.00	52.00	Data not available	Data not available	0.70
5	14.83	41.08	42,000	5,800	27.60	59.40	Data not available	Data not available	0.90
6	11.00	Data not available	Data not available	3,260	43.00	57.00	Data not available	Data not available	Data not available
7	10.40	32.00	118,000	3,220	25.40	68.30	Data not available	189	1.10
8	7.97	25.78	25,000	5,080	36.60	47.20	21	31	1.04
9	11.17	34.20	47,000	2,500	50.40	29.10	23	29	0.90
10	9.30	28.20	69,000	4,200	49.00	35.60	49	28	0.50
11	9.99	29.00	101,000	3,760	24.00	62.00	25	21	0.70
12	15.00	Data not available	105,000	8,500	6.00	94.00	Data not available	Data not available	1.00
13	10.00	34.40	43,000	7,960	43.00	44.70	Data not available	Data not available	Data not available
14	6.51	18.88	317,000	6,070	69.40	12.50	Data not available	Data not available	1.00
15	11.36	35.14	150,000	10,800	66.00	25.20	Data not available	Data not available	Data not available
16	6.20	18.10	328,000	8,000	34.80	58.50	10	25	0.80

### Treatment received during hospitalization

All prescribed medication was available for use by medical staff, and all medication administered was in accordance with the prescribed scheme. Anti-malarial treatment regimes consisted primarily of primaquine 93.8% (15/16) and chloroquine 87.5% (14/16), followed by clindamycin 18.3% (3/16), artemether-lumefantrine 12.5% (2/16), quinine 6.3% (1/16), and artesunate 6.3% (1/16). Doxycycline was not used in any of the cases. 87.5% (14/16) of cases received oral medication, while 12.5% (2/16) received treatment intravenously, after which they were switched to oral treatment. The median number of days with intravenous medication was five (range: 4–6 days), while the median number of days on oral treatment was seven days (range: 7–20 days). Physical mediums to reduce fever were used in all cases.

### Hospitalization data

The median number of days hospitalized was four (range: 1–8 days), 25% (4/16) of cases were treated in the intermediate care unit. Intermediate care unit cases had a higher median number of days hospitalized of 4.5 days, and ranged between four and six days hospitalized.

### Complications developed during hospitalization

None of the cases presented haemoglobinuria, respiratory difficulty, altered mental status, prostration, seizures, inability to feed or hyperparasitaemia. Hyperlactataemia, metabolic acidosis and pulmonary oedema were not reported in the medical records, there was no evidence of arterial blood gases or chest x rays.

Nine from the 16 cases presented anaemia during hospitalization of which 22.2% (2/9) had severe anaemia, and 33.3% (3/9) received transfusion of red blood cells. Jaundice was presented in five out of 16 cases; it was consider a severity sign in three cases due to the presence of jaundice plus signs of organ dysfunction. Further information about complication developed during hospitalization is summarized in Table 5.

**Table 5:** **Frequency of complications presented on admission or during hospitalization**

Complications	n	%
Spontaneous bleeding	2	12.50
Shock: Systolic pressure <80 mm/Hg (adults), <50 mmHg (children)	3	18.57
Severe normocytic anaemia: Haemoglobyn <7 g/dL, hematocryte <21%	2	12.50
Hypoglaecemia <60 mg/dL	1	6.25
Acute renal failure: Creatinine >1.5 mg/dL	1	6.25
Elevated aminotransferase >80 U.I.	3	18.75
Hyperbilirubinemia: Total bilirubin >3 mg/dL	6	37.50
Thrombocytopaenia <50,000 mm3	4	25.00
Jaundice with evidence of organ dysfunction	3	18.57
**One complication (8/16; 50.00%)**		
**More than one complication (8/16; 50.00%)**		

## Discussion

Information from medical records of sixteen cases of severe vivax malaria hospitalized in the municipalities of Tumaco, Cali, and Buenaventura, between 2009 and 2013 was collected retrospectively. The majority of the cases originated from Tumaco, one of the cases presented comorbidities that didn’t seem to affect its outcome. Principal symptoms found at time of admission were fever, chills, and headache. Main complications found were: hyperbilirubinaemia, thrombocytopaenia and jaundice with evidence of organ dysfunction. Most of the cases received Chloroquine and primaquine as main treatment.

The Pacific Colombian geographic region has socio-economic and environmental conditions able to the transmission of malaria species as described by Arevalo. et al. [8, 11]. *Plasmodium vivax* is the most prevalent malaria parasite in Colombia, even though *P. falciparum* has been described as the main cause of severe malaria and malaria caused by *P. vivax* has been considered a benign condition, this outlook has been challenged in the last decade, and complications have been described [18, 30].

According to the literature, cases that had malaria and one or more co-morbidities tended to develop more complications and severe presentations [24, 26, 31]. From the 16 cases described, only one case was diagnosed with arterial hypertension, diabetes mellitus and cerebrovascular disease. Despite the presence of co-morbidities, no evidence of any particular pattern of increased complications during hospitalization was found.

The symptoms that were mentioned by cases were concordant with symptoms most frequently mentioned in medical literature, such as fever, chills, headache, musculoskeletal pain, asthenia, and adynamia [8]. Respiratory and gastrointestinal symptoms were not as frequently mentioned by theses cases. Presence of pruritus was exclusively reported in pregnant cases, as an incidental symptom.

Of particular interest was the presence of hepatomegaly in four cases, jaundice in five cases, elevated transaminase in three cases, and elevated bilirubin values in six cases, which had been reported in the literature as common signs and laboratory values associated with a complication of vivax malaria [7, 21, 27, 30, 32].

With regard to vital signs, it was observed that the median body temperature was 37.0°C, which is lower than expected value for a case with malaria. This may confuse diagnosis and delay treatment. Although there were only three cases that reported self-medication, data was not available for almost half of the cases (7/16); it is possible that antipyretic use prior to consultation masked the fever. A certain degree of immunity in endemic areas has also been suggested as cause of afebrile infection and low severity of symptoms [8].

Malaria diagnosis was suspected through signs and symptoms mentioned above, to confirm the diagnosis all cases underwent paraclinical evaluation through blood smear. Thick blood smear was used in 15 cases, while thin blood smear was used in one case. These methods are widely available in endemic regions in Colombia due to their low costs, despite the existence of more sensitive and specific diagnostic tests [29, 33].

Additional paraclinical data were registered to complement the medical evaluation and asses malaria severity. Anaemia, thrombocytopaenia, hepatic dysfunction, acute renal failure, hypoglycemia were evidenced.

Severe anaemia is described in literature as one of the main severity complications of endemic regions [8, 32]. Anaemia was found in nine of the 16 cases, two of them were considered severe anaemia (haemoglobin <7 mg/dL) and required blood transfusion.

Given the socio-economic conditions of the population, it is difficult to associate anaemia solely with *P. vivax*, as other factors, such as malnutrition, could have influenced the presence of anaemia [34–36]. Currently the literature on this topic in Colombia is scarce, or focused in children and thus should be studied.

Low platelet count is been considered a common complication presented in severe vivax malaria, and causal factor of mucosal bleeding [37]. 25% of the cases had a count below 50,000 mm3 and only two cases reported haemorrhagic manifestations. Renal damage was found in one of the cases included in the study; creatinine levels were slightly above normal range, another explanation for this increment could be dehydration.

None of the cases presented signs of pulmonary oedema or acute respiratory distress syndrome, therefore, there was no indication to order chest X ray to rule out the complication. Müller et al. consider the low rate of pulmonary signs to be a result of cases residing in an endemic region, where they develop protective immunity, reducing these pulmonary complications [7, 27].

Hepatic dysfunction, represented principally as an elevation of total bilirubin value, was the foremost salient criterion for diagnosis of severe vivax malaria. As described in the literature, elevation of bilirubin has been associated with anaemia and thrombocytopaenia [27, 29, 38]. In this study 25% of cases presented anaemia and elevated bilirubin levels.

According to the World Health Organization (WHO) international guidelines [39], hepatic complication was present in two cases; one case had total bilirubin levels above 3 mg/dl, and had a three fold elevation of transaminase normal value while the other case only presented total bilirubin levels greater than 3 mg/dl.

In agreement with the literature, the WHO criteria are too demanding, resulting in a low detection sensitivity [28, 40]. Additionally, the national guideline criteria of abnormal hepatic dysfunction are 3,5 times more sensitive than the WHO criteria. For this reason, it was decided to utilize an intermediate value for transaminase values of 80 IU to classify abnormal hepatic dysfunction [28]. No cases presented with acute hepatic failure as defined by King’s College criteria [41].

With regard to treatment, all prescribed medications were available and administered in a timely manner. In Colombia, the first-line treatment for non-severe malaria is chloroquine and primaquine. Despite reports of resistance since 2001, it appears to be effective for vivax infection [42–44]. The first-line treatment for severe malaria is intravenous artesunate, followed by combined oral treatment artemether and lumefantrine. Pregnant women should receive combined treatment with quinine and clindamycin [42].

Chloroquine and primaquine were the most prescribed medications, 93.8% (15/16) and 87.5% (14/16), respectively, which suggests that the cases are being treated as non-severe malaria. Clindamycin was prescribed in three cases and quinine was prescribed in one case; the two pregnant cases did not receive medication indicated by national guidelines. Artemether-lumefantrine was only prescribed for two cases and only one of the cases received artesunate. All cases received antipyretic and crystalloids during hospitalization. Other forms of medication received included gastric mucosal protective agents, followed by analgesics and then diuretics.

Despite chloroquine and primaquine not being recommended in national guidelines for severe *P. vivax* cases, there was a notable reduction of symptoms and disease progression, which was related to a positive outcome in all cases.

Limitations of the study include quantity, distribution of cases obtained, which did not represent the established rates of infection in Colombia or reported vector behaviour. The retrospective nature of the design of the study used to collect data was a limitation within itself. Most of the medical records reviewed were handwritten; only in 2013 were the majority of visited hospitals beginning to utilize electronic medical records. This limited the mining of data due to the high proportion of incomplete, fractionated or illegible medical records before 2013, thus explaining in part the concentration of data in 2013.

In agreement with Tobón, et al., the fact that cases seek medical attendance at different stages of the disease could imply that some symptoms of severity might be present while others are not at the time of admission [27]. This could have led to different approaches towards case diagnosis, treatment and paraclinical examination possibly explaining the disparity in data between cases.

## Conclusion

This retrospective case-series of 16 hospitalized cases of vivax malaria was written to contribute to the knowledge regarding the behaviour and clinical expression of this disease. A predominance of fever, chills and headache as admission symptoms was evidenced, and an elevation of total bilirubin levels, and severe thrombocytopaenia were the main complications presented during hospitalization. All cases responded to treatment, there were no deaths. Questions arising from this study that can be followed up to include: 1) If the time to consultation has an impact on the number of days hospitalized and how cases progress during hospitalization 2) If the severity criteria in WHO guidelines is sensitive enough to be used in clinical practice, versus national guidelines, and 3) If there is a particular role that malnutrition contributes to in malaria-endemic regions that suffer from anaemia.

## Abbreviations

ICU: Intermediate care units
BUN: Blood urea nitrogen
WHO: World Health Organization
ALT: Alanine aminotransferase
AST: Aspartate aminotransferase
BPM: Beats per minute
RPM: Respirations per minute
IU: International units.
